# Evolution and Dynamics of Regulatory Architectures Controlling Polymyxin B Resistance in Enteric Bacteria

**DOI:** 10.1371/journal.pgen.1000233

**Published:** 2008-10-24

**Authors:** Alexander Y. Mitrophanov, Mollie W. Jewett, Tricia J. Hadley, Eduardo A. Groisman

**Affiliations:** 1 Howard Hughes Medical Institute, Washington University, St. Louis, Missouri, United States of America; 2 Department of Molecular Microbiology, Washington University, St. Louis, Missouri, United States of America; University of Toronto, Canada

## Abstract

Complex genetic networks consist of structural modules that determine the levels and timing of a cellular response. While the functional properties of the regulatory architectures that make up these modules have been extensively studied, the evolutionary history of regulatory architectures has remained largely unexplored. Here, we investigate the transition between direct and indirect regulatory pathways governing inducible resistance to the antibiotic polymyxin B in enteric bacteria. We identify a novel regulatory architecture—designated feedforward connector loop—that relies on a regulatory protein that connects signal transduction systems post-translationally, allowing one system to respond to a signal activating another system. The feedforward connector loop is characterized by rapid activation, slow deactivation, and elevated mRNA expression levels in comparison with the direct regulation circuit. Our results suggest that, both functionally and evolutionarily, the feedforward connector loop is the transitional stage between direct transcriptional control and indirect regulation.

## Author Summary

A regulatory protein can activate the expression of a target gene either directly, i.e., by binding to the gene's promoter, or indirectly, i.e., by altering the expression of regulators, which, in turn, bind to the target gene's promoter and induce or inhibit its transcription. Indirect regulatory circuits can contain multiple components and functional elements, such as feedforward and feedback loops. The complex structure of indirect regulation raises the question of its evolutionary origins. Here, we study the dynamic and evolutionary properties of regulatory architectures that involve members of the recently emerged class of bacterial proteins termed connectors. Such proteins post-translationally modulate the activity of two-component systems and phosphorelays, which constitute the prevalent form of bacterial signal transduction. We describe a novel connector-mediated regulatory circuit that combines the structural and functional properties of direct and indirect regulation. Our results indicate that this architecture is the evolutionary link between direct and connector-dependent regulatory designs.

## Introduction

Related organisms often express orthologous genes in response to a particular cellular or environmental cue. However, the regulatory mechanisms promoting expression of these genes can be drastically different, ranging from direct transcriptional control to multi-stage architectures involving feedback loops, feedforward loops and regulatory cascades [1]–[5]. Extensive studies of the functional properties of recurrent regulatory architectures–termed network motifs–have revealed that they exhibit quantitative differences in the levels and timing of gene expression [1]. Whereas the dynamical properties of distinct network motifs are relatively well understood, there is still limited knowledge about the general principles underlying the quantitative features and evolutionary relationships of genetic regulatory architectures.

A prevalent form of bacterial signal transduction is the two-component system and its more complex version, the phosphorelay [6]–[9]. The activity of two-component systems and phosphorelays can be modulated at the post-translational level by members of the recently emerged class of proteins designated connectors (reviewed in [10]), which modulate the output of a two-component system in response to signals other than the ones directly sensed by the system. In addition to facilitating signal integration, connectors confer specific quantitative properties on the regulated systems, which could result in survival advantages for the bacterium [2].

**Figure 1:**
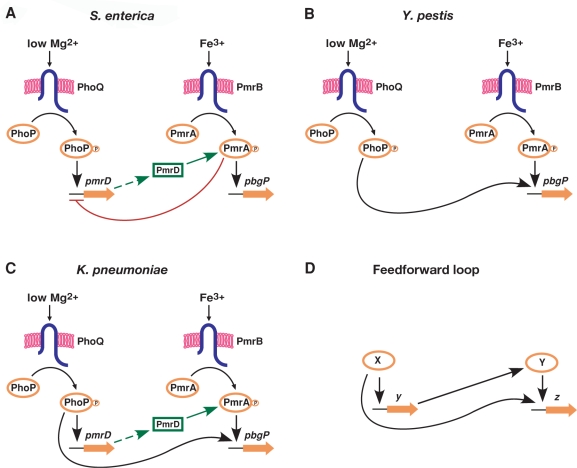
Regulatory architectures controlling expression of the polymyxin B resistance operon *pbgP* in different bacterial species. Also shown is a schematic of the feedforward loop architecture. (A) The connector-mediated pathway activating the *pbgP* operon in *S. enterica*. Transcription of *pbgP* is promoted during growth in low Mg^2+^ via the PhoP/PhoQ system, PmrD protein and the PmrA/PmrB system; in the presence of Fe^3+^, it is promoted via the PmrA/PmrB system independently of PhoP/PhoQ and PmrD. The PmrA protein represses transcription of the *pmrD* gene. (B) The direct activation pathway promoting *pbgP* transcription in *Y. pestis*. Transcription of the *pbgP* gene is promoted during growth in low Mg^2+^ directly via the PhoP/PhoQ system, and in the presence of Fe^3+^ directly via the PmrA/PmrB system. The *pmrD* gene is absent from the *Y. pestis* genome (Figure 2). (C) The feedforward connector loop activating *pbgP* in *K. pneumoniae*. Transcription of the *pbgP* gene is promoted during growth in low Mg^2+^ via the PhoP/PhoQ system, the PmrD protein and the PmrA/PmrB system, as well as directly by the PhoP protein binding to the *pbgP* promoter. In the presence of Fe^3+^, transcription is activated directly by the PmrA/PmrB system, independently of PmrD and PhoP/PhoQ. (D) The feedforward loop. A transcriptional regulator X controls gene *z* both directly and indirectly, via an additional transcriptional regulator Y that activates gene *z*.

The best characterized connector-dependent pathway is mediated by the PmrD protein (NCBI protein database accession number AAL21205) in the bacterium *Salmonella enterica* serovar Typhimurium, where it enables expression of genes controlled by the PmrA/PmrB two-component regulatory system in response to the low Mg^2+^ signal that activates the PhoP/PhoQ system [2], [11]–[13] (Figure 1A). PmrD is a PhoP-activated protein that binds to the phosphorylated form of the DNA binding regulatory protein PmrA (PmrA-P), thereby protecting it from dephosphorylation by PmrA's cognate sensor PmrB [11]. This results in binding of PmrA-P to its target promoters and in changes in transcription of the corresponding genes such as *pbgP* (also referred to as *pmrH* [14] and *arnB* [15]), which mediates a chemical modification in the lipopolysaccharide that confers resistance to the antibiotic polymyxin B [16]–[18]. This architecture allows *S. enterica* to express PmrA-activated genes and to display polymyxin B resistance in response to the signals activating the PhoP/PhoQ system [19] as well as in the presence of Fe^3+^, Al^3+^ or acid pH, which are specific activating signals sensed by PmrB [20],[21]. Expression of PmrA-dependent genes is slightly reduced in a *pmrD* mutant when both inducing signals, low Mg^2+^ and Fe^3+^, are present [2], [11]–[13].

The related enteric species *Yersinia pestis* also promotes *pbgP* expression and demonstrates polymyxin B resistance in response to Fe^3+^ and/or low Mg^2+^, even though it lacks *pmrD* [22]. This is because the *Y. pestis pbgP* promoter harbors binding sites for both the PhoP and the PmrA proteins [22] (referred to as PhoP and PmrA boxes, respectively) (Figure 1B). A comparison of the *Yersinia*-like direct transcription regulation circuit, which was reconstructed in an engineered *S. enterica* strain, to the connector-mediated pathway of wild-type *S. enterica* demonstrated that the latter pathway exhibits heightened induction ratios, which results in increased levels of polymyxin B resistance [2]. The connector-mediated pathway also displayed slower expression induction and increased persistence of expression after a shift from inducing to repressing conditions in comparison with the direct activation pathway [2]. Persistence of expression may facilitate the continuous synthesis of the PmrA-dependent cell envelope modifying determinants in fluctuating environments [2].

In this paper, we identify a novel regulatory architecture that combines structural and functional features of the direct regulation circuit and the connector-mediated pathway. The novel architecture, termed feedforward connector loop, possesses a direct regulatory branch, like that in *Y. pestis*, and an indirect branch that is analogous to the connector-mediated pathway of *S. enterica*. Even though the simultaneous presence of direct and indirect branches of regulation also characterizes one of the most abundant network motifs (*i.e.*, the feedforward loop) [1],[3], the identified architecture demonstrates substantial differences in dynamical behavior. Analysis of several enteric species suggests that the feedforward connector loop is the evolutionary link between direct transcriptional control and the connector-mediated regulatory circuit.

## Results

### *K. pneumoniae* Harbors a PhoP-Activated *pmrD* Ortholog

**Figure 2:**
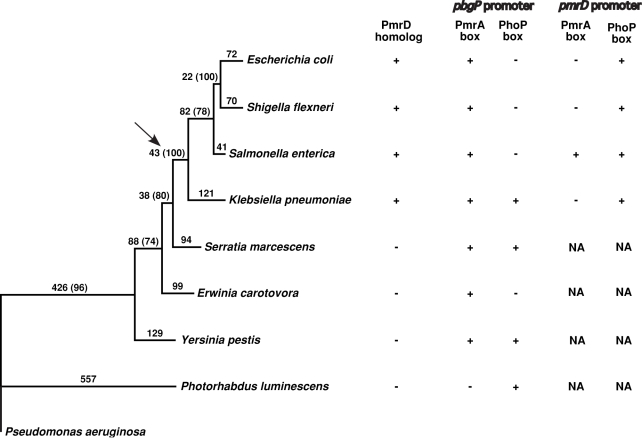
Phylogenetic relationship of the analyzed bacterial species harboring the polymyxin B resistance *pbgP* operon. The phylogenetic tree was constructed as described in Materials and Methods. Numbers on the branches refer to branch lengths (which indicate the numbers of amino acid substitutions along the corresponding branches), and numbers in the parentheses represent bootstrap support (which indicates the percentage of the replicates from random resampling of the data where a particular node is found in the best tree). The solid arrow indicates the putative acquisition of the *pmrD* gene. The signs “+” or “−” denote, respectively, presence or absence of a protein ortholog or a transcription factor binding site. NA—not applicable.

**Figure 3:**
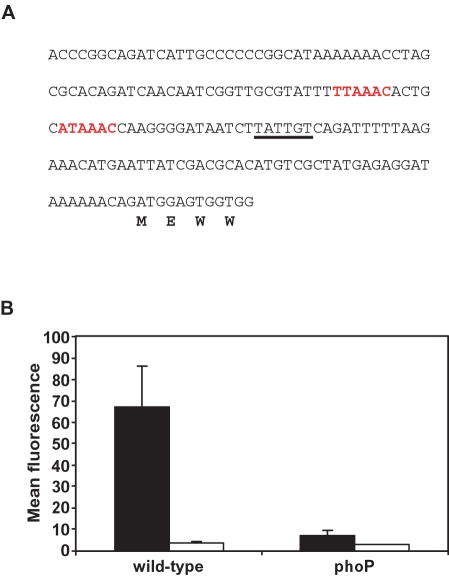
Expression of the *K. pneumoniae pmrD* gene is promoted in low Mg^2+^ in a PhoP-dependent manner. (A) DNA sequence of the promoter region of the *K. pneumoniae pmrD* gene. The putative PhoP box [22] is in red and the putative −10 region is underlined. The first four amino acid residues of the *pbgP* ORF are indicated below the nucleotide sequence. (B) Fluorescence of a *pmrD-gfp* transcriptional fusion was measured in wild-type (EG13127) and isogenic *phoP* (EG15289) *K. pneumoniae* strains harboring the pAG-*pmrD_Klebsiella_* plasmid following growth in N-minimal medium, pH 7.7, containing 38 mM glycerol with either 10 µM (black bars) or 10 mM (white bars) Mg^2+^ as described in Materials and Methods. Strains harboring the control pAG-*rpmS* plasmid and the pAG vector demonstrated constitutive fluorescence and no fluorescence in all growth conditions, respectively (data not shown).

To explore the potential evolutionary scenario responsible for the PmrD-mediated architecture, we analyzed the distribution of the *pmrD* gene, and of PhoP and PmrA boxes in the *pmrD* and *pbgP* promoters among enteric bacteria (Figure 2). We looked for a bacterial lineage displaying evidence for both connector-mediated (Figure 1A) and direct (Figure 1B) regulation of the *pbgP* operon. *K. pneumoniae* appeared to fit these criteria because its genome harbors a *pmrD* ortholog (Figure S1) that is preceded by a PhoP box (Figure 3A), and because sequences resembling PhoP and PmrA boxes were present upstream of the *pbgP* operon (Figure 2).

We tested the genomic prediction that the *K. pneumoniae pmrD* gene is PhoP-activated by investigating *pmrD* transcription in wild-type, *phoP* and *pmrA* strains grown under different conditions. The *pmrD* gene was expressed during growth in low Mg^2+^ in a PhoP-dependent manner but not in high Mg^2+^ (Figure 3B), like the *S. enterica* [13] and *E. coli* [23] orthologs. In contrast to what happens in *S. enterica*, *pmrD* transcription was not repressed by the PmrA protein in *K. pneumoniae* (Figure S2), consistent with the absence of sequences resembling a PmrA box in the *pmrD* promoter region (Figure 3A).

### A Novel Regulatory Architecture Controls *pbgP* Expression in *K. pneumoniae*

**Figure 4:**
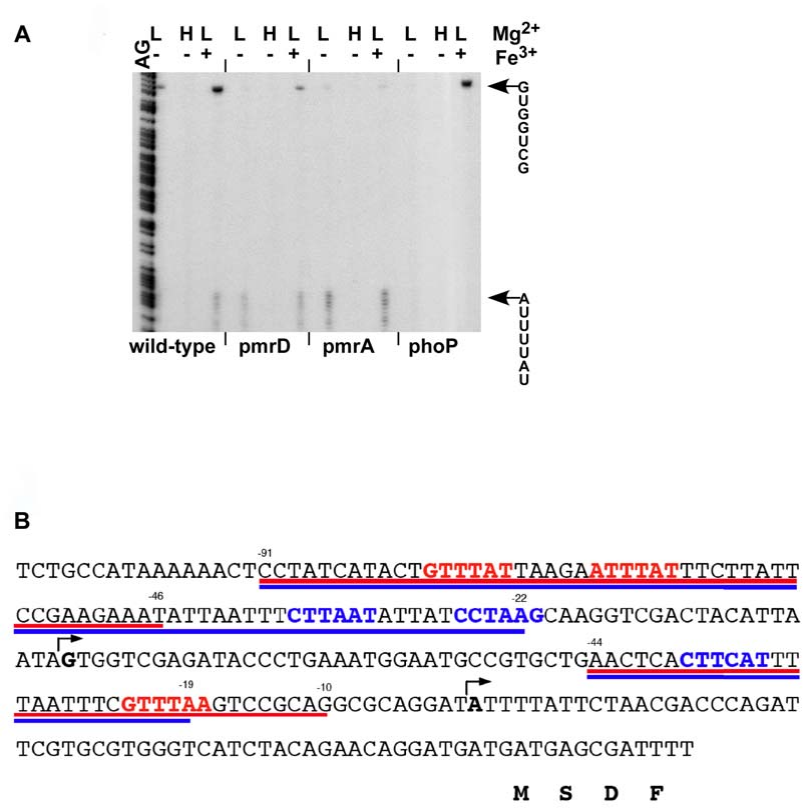
Dependence of *pbgP* transcription on the *pmrA*, *pmrD*, and *phoP* genes under different growth conditions. (A) S1 nuclease-protection assay of RNAs extracted from bacteria grown at 37°C in N-minimal medium, pH 7.7, with 10 µM MgCl_2_ (L, −), 10 mM MgCl_2_ (H, −), or 10 µM MgCl_2_ and 100 µM FeSO_4_ (L, +). Lane AG corresponds to the Maxam-Gilbert DNA ladder of the target sequence. The sequences spanning the two transcription start sites are shown, and the start sites are indicated with arrows. (B) DNA sequence of the promoter region of the *K. pneumoniae pbgP* gene. The two transcription start sites are indicated in bold and with arrows. The sequence in blue resembles the PmrA-box consensus [22], the sequence in red resembles the PhoP-box consensus [22]. The DNA sequence underlined with blue and red indicates the regions footprinted by the *S. enterica* PmrA and PhoP proteins, respectively (the footprinting data are shown in Figure S3). The first four amino acid residues of the *pbgP* ORF are indicated below the nucleotide sequence.

To define the regulatory circuit governing *pbgP* transcription in *K. pneumoniae*, we investigated *pbgP* transcription in isogenic wild-type, *pmrA*, *phoP* and *pmrD* strains grown under different conditions promoting activation of the PhoP/PhoQ and PmrA/PmrB systems. S1 mapping experiments revealed two transcription start sites for the *pbgP* gene in wild-type *K. pneumoniae*: an ORF-proximal site that was active upon growth in low Mg^2+^ or in low Mg^2+^+Fe^3+^, but not in high Mg^2+^; and an ORF-distal site that displayed higher activity in low Mg^2+^+Fe^3+^ than in low Mg^2+^ (Figure 4A). The ORF-proximal promoter was PhoP-dependent but PmrA- and PmrD-independent, whereas the ORF-distal promoter was induced in low Mg^2+^ in a PhoP-, PmrD- and PmrA-dependent fashion, and in the presence of Fe^3+^ in a PmrA-dependent but PhoP- and PmrD-independent manner. DNase footprinting experiments with the conserved PhoP and PmrA proteins from *S. enterica* demonstrated specific binding to the *K. pneumoniae pbgP* promoter at the predicted PhoP and PmrA boxes (Figure 4B and Figure S3), indicating that the PhoP and PmrA proteins exert their regulatory effects directly. This regulatory architecture, in which PhoP activates *pbgP* expression directly by binding to the *pbgP* promoter, and indirectly via PmrD-dependent activation of the PmrA protein also binding to the *pbgP* promoter, was designated *f*eedforward *c*onnector *l*oop (or FCL) (Figure 1C) because it resembles the feedforward loop [3] network motif [1].

### Mathematical Modeling of the Feedforward Connector Loop

The feedforward loop (FFL) is one of the most abundant network motifs in prokaryotic regulatory circuits [1],[3],[24]. In a FFL, a transcriptional regulator X controls expression of gene *z* both directly, by binding to its promoter region, and indirectly, by promoting expression of gene *y* encoding a transcriptional regulator Y that also binds to the promoter of gene *z* (Figure 1D). FFLs exhibit special functional features [1],[3],[25], including the ability to act as sign-sensitive delay elements: they can increase the time it takes to activate gene expression while keeping the deactivation time unaffected, or the other way around [3],[26],[27]. For example, the coherent, activation-type FFL with an OR-gate can promote deactivation delays when compared to a circuit with direct regulation, though activation times for both designs are similar [3],[26].

Regulation by the FCL architecture identified in *K. pneumoniae* (Figure 1C) is qualitatively equivalent to regulation by the latter type of the FFL, because the FCL follows the OR type of logic (Figure 4A). Yet, the FCL differs from the FFL in that, instead of a two-stage transcriptional activation cascade, it relies on one transcription factor (*i.e.*, PhoP) to promote expression of a connector protein (*i.e.*, PmrD) that activates another transcription factor (*i.e.*, PmrA) at the post-translational level (Figure 1C, D) [3],[26].

**Figure 5:**
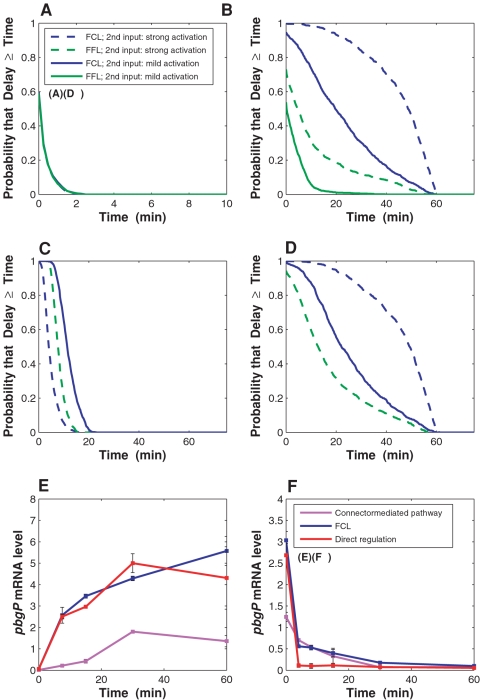
Activation and deactivation dynamics of connector-mediated and direct regulatory circuits. Delay length distributions for the feedforward connector loop (FCL) and feedforward loop (FFL) ((A)–(D)) and experimental measurements of activation and deactivation dynamics for the connector-mediated pathway, FCL, and direct regulation circuit ((E)–(F)). In the simulations, the second input with strong and mild activation corresponds to high and low ratios of phosphorylation/dephosphorylation rates for PmrA (or for protein Y of the FFL), respectively. In the activation and deactivation experiments, the connector-mediated pathway was harbored by the wild-type *S. enterica* strain (14028s) whereas the FCL and direct regulation circuit were harbored by the engineered *S. enterica* strains EG17353 and EG17354, respectively. mRNA levels were determined as described in Materials and Methods. (A) Activation delay length distributions for the FCL and FFL. (B) Deactivation delay length distributions for the FCL and FFL. (C) Activation delay length distributions for the FCL and FFL lacking direct activation branches. (D) Deactivation delay length distributions for the FCL and FFL lacking direct activation branches. (E) Activation dynamics for *pbgP* gene expression in *S. enterica*. (F) Deactivation dynamics for *pbgP* gene expression in *S. enterica*.

To define the salient characteristics of the FCL architecture, we analyzed activation and deactivation times, and contrasted these properties to those of the direct regulation circuit, the connector-mediated pathway, and the FFL. We utilized a variety of parameter values with a mathematical modeling methodology that was successfully used in the comparative analysis of the connector-mediated and direct regulation pathways [2] (see Materials and Methods). In our computations, the PhoP-P level (determined by the abundance of Mg^2+^ in the extracellular environment) was the main input for the regulatory circuits. An additional input was the level of PmrA-P, which reflects the activity of the PmrA/PmrB system (stimulated by Fe^3+^); in the FFL case, the second input was the level of activated (phosphorylated) protein Y (Figure 1D). For this second input, we considered the cases of mild and strong activation. The case of mild activation of the second input for the transcriptional cascade was not considered because when the second input is inactive, two-component systems connected by a transcriptional cascade cannot be activated [28] (Figure 5C, D: no green solid lines).

The FCL and the FFL displayed an equivalent ability to promote small activation delays with respect to the direct regulation circuit (Figure 5A). Whereas the FFL promoted large deactivation delays only with a small probability, large deactivation delays in the FCL could be observed in a substantial fraction of the cases (Figure 5B). The FCL acted as a true sign-sensitive delay element for most of the simulated parameter values, but the FFL did not (Figure 5 and Figures S4, S5, and S6). Therefore, the FCL architecture generally provides much stronger sign-sensitive delay elements than the FFL design.

Models for the connector-mediated pathway and a two-stage transcriptional cascade (corresponding to the FCL and FFL with the direct regulation branches removed, respectively) possessed a high ability to promote both activation and deactivation delays (Figure 5C, D; Figures S4, S5, and S6), in agreement with experimental data [2],[5],[29]. Notably, deactivation delay distributions for the FCL and the connector-mediated pathway in the case of strong activation of the second input are nearly identical (Figure 5B, D; Figures S4B, D, S5, and S6B, D). This allows us to conclude that, when the second input is strong (which leads to elevated PmrA-P level and, therefore, heightened induction of the connector-mediated branch of regulation), the deactivation delays are determined almost entirely by the connector-mediated branch. A mathematical comparison of model outputs suggested that the FFL and FCL give higher output levels than their counterparts lacking direct activation branches (Equation 16 in Text S1). This can be ascribed to the presence of an additional branch of *pbgP* regulation which would increase the proportion of active *pbgP* promoters, leading to a higher production rate for the *pbgP* mRNA.

### The FCL Dynamics Demonstrate Sign-Sensitive Delays in Gene Expression

To test the modeling predictions regarding the timing and output levels of *pbgP* expression in the different architectures (Figure 5A, B, C, D; Equation 16 in Text S1), we measured the *pbgP* mRNA levels in isogenic *S. enterica* serovar Typhimurium strains harboring the connector-mediated pathway (Figure 1A), or engineered to express *pbgP* utilizing the direct regulation circuit (Figure 1B) or the FCL (Figure 1C). This allowed us to focus on the quantitative features determined by the circuit architecture (as opposed to its specific implementation in a particular species), and to avoid comparison biases arising from the inherently distinct biology of different bacterial species [1]. This is consistent with the previously established genetic circuit comparison methodology [2].

Our computational analysis showed that the connector-mediated pathway typically displays activation delays (when compared to the direct regulation circuit) whereas the FCL does not (Figure 5A, C), suggesting that *pbgP* expression would be activated sooner in the strain with the FCL than in the one with the connector-mediated pathway. Indeed, when cells were grown under non-inducing conditions (*i.e.*, 10 mM Mg^2+^) for 4 h and then switched to inducing conditions (*i.e.*, 20 µM Mg^2+^) at time 0, the *pbgP* mRNA level rose much faster in the FCL than in the connector-mediated pathway (Figure 5E). (Activation and deactivation affected only the PhoP-dependent input of the circuits through changes in the Mg^2+^ concentration, because there was no direct PmrA activation input due to the absence of Fe^3+^ in the medium.) This rapid activation was ascribed to the direct regulation branch because the connector-mediated pathway, which lacks a direct regulation branch (Figure 1A), displayed delayed activation (Figure 5E) [2]. Furthermore, the direct regulation circuit (in a similar way to the FCL) demonstrated rapid activation (Figure 5E).

For the case of deactivation, our computations predicted that the FCL and the connector-mediated pathway typically generate a delayed deactivation response compared to the direct regulation circuit (Figure 5B, D). When cells were grown for 2 h in a medium containing 20 µM Mg^2+^ and then switched to non-inducing conditions at time 0, deactivation was notably slower in the FCL than in the direct regulation circuit and was correlated with the expression persistence displayed by the connector-mediated pathway (Figure 5F). These results are in agreement with the previously obtained experimental data on the connector-mediated pathway dynamics [2]. Finally, the output levels promoted by the FCL were generally higher than those for the connector-mediated pathway (Figure 5E, F), consistent with our theoretical prediction regarding the contribution of two positive regulation branches (Equation 16 in Text S1).

## Discussion

The level at which a gene is transcribed depends on the *cis* features of the gene promoter, which govern its interactions with RNA polymerase and regulatory proteins, as well as on the architecture that determines the levels and activity of these proteins. We have identified a novel regulatory architecture–termed FCL–that mediates activation of the polymyxin B resistance gene *pbgP* by the PhoP protein in *K. pneumoniae*. The FCL is characterized by two branches of regulation: a direct branch where the PhoP protein directly promotes *pbgP* transcription by binding to the *pbgP* promoter, and an indirect branch in which the PhoP-dependent PmrD protein activates the PmrA protein, which, in turn, binds to the *pbgP* promoter. The FCL structure was inferred from the following findings. First, expression of the connector protein PmrD is activated in low Mg^2+^ in a PhoP-dependent fashion. Second, the PhoP-mediated activation of *pmrD* transcription appears to be direct because the *pmrD* promoter harbors a PhoP box (Figure 3A). Third, growth in low Mg^2+^ activates two *pbgP* promoters: one that is PhoP-dependent, but PmrA- and PmrD-independent, and another one that is PhoP-, PmrA-, and PmrD-dependent (Figure 4A). And fourth, the PhoP and PmrA proteins bind to the *pbgP* promoter region (Figure 4B and Figure S3). The FCL may represent an intermediate stage between direct control (Figure 1B) and the connector-mediated pathway (Figure 1A).

From the point of view of regulatory logic, the FCL would appear to be a redundant circuit because any one of the two activation branches is sufficient to promote *pbgP* expression (Figure 4A). Such a “redundancy” also characterizes the FFL (Figure 1D), one of the most abundant network motifs identified in bacteria [1],[3],[24]. However, the presence of an extra branch of regulation confers special dynamic properties on these two designs. The FCL acts as a sign-sensitive delay element, promoting large deactivation delays but no (or very small) activation delays (Figure 5A, B, E, F). The ability of the FCL to promote sign-sensitive delays can be explained by its architecture (Figure 1C). Fast activation is due to the presence of a direct activation branch (as in a direct regulation circuit (Figure 1B)), which distinguishes the FCL from the connector-mediated pathway exhibiting longer activation delays associated with the necessity to synthesize the PmrD protein (Figure 5C, E) [2]. At the same time, the indirect branch of the FCL guarantees *pbgP* expression persistence upon deactivation (Figures 5, S4, S5, and S6), which, as with the connector-mediated pathway [2], is likely due to the PmrD protein made before the cells were switched to non-activating conditions. In addition, our results indicate that the FFL promotes only relatively small deactivation delays, which is in contrast to the large delays that are typical of the FCL (Figure 5B). The presence of two branches of activation in the FCL results in higher *pbgP* expression levels compared with the connector-mediated pathway (Equation 16 in Text S1; Figure 5E, F). Additional insights into the functionality of the FCL might be provided by dynamics studies in the stochastic (single-cell) setting [30] as demonstrated for the FFL [25].

The discovery of the novel PmrD-mediated architecture–the FCL–suggests a plausible parsimonious scenario for the evolution of Mg^2+^-dependent polymyxin B resistance in enteric bacteria. First, the *Klebsiella* and *Salmonella* lineages diverged after their common ancestor had split from the *Yersinia* lineage (Figure 2). Second, PmrD homologs are present in all species derived from this common ancestor, but in none of the remaining species (Figure 2). And third, the *pbgP* promoter of *Serratia marcescens*, which is a close relative of the immediate ancestor of *Klebsiella*, harbors both PhoP and PmrA boxes (Figure 2). It is thus conceivable that the *pmrD* gene was “invented” or horizontally acquired by the common ancestor of *Salmonella*, *Klebsiella*, *Shigella*, and *Escherichia* [31]–[33]. After diverging from the *Klebsiella* lineage, the ancestral lineage of *Salmonella*, *E. coli* and *Shigella* would have lost the direct branch of *pbgP* activation by the PhoP protein, as none of these species harbor a PhoP box in the *pbgP* promoter.

The hypothesized transition from the FCL design utilized by *K. pneumoniae* to the connector-mediated pathway operating in *S. enterica* might have obeyed the need to avoid overproduction of PmrA-activated gene products. Indeed, hyperactivation of the PmrA/PmrB system can have detrimental effects, such as increased susceptibility to the detergent deoxycholate [34] and to the antimicrobial peptide protamine (E. A. Groisman, unpublished results). Apparently, this need had a substantial influence on the connector-mediated pathway as *S. enterica* evolved a negative feedback loop to repress PmrD production [12], thereby preventing excessive expression of PmrA-activated genes. The activation delays, which result from elimination of the direct regulation branch, are in the case of *S. enterica* relatively small [2]. Thus, the circuit's responsiveness, while somewhat decreased, appears to be sufficient for survival in the specific niche occupied by this bacterium.

The evolution of connector-mediated pathways is driven by changes both in the connector protein genes and in the transcriptional regulatory interactions. Genes encoding connectors can undergo rapid sequence and functional divergence, resulting in novel regulatory architectures. For example, diversifying selection on the PmrD protein has resulted in the majority of *E. coli* natural isolates lacking the ability to express PmrA-activated genes in response to the signals activating the PhoP/PhoQ system [23]. Likewise, the divergence of the *iraP* promoter sequence between *S. enterica* and *E. coli* results in the inability of the *E. coli* connector IraP to inhibit the degradation of the alternative sigma-factor RpoS in low Mg^2+^, whereas the *S. enterica* IraP performs this function because it is produced under such conditions [35].

Bacterial genetic regulatory circuits are shaped by the properties of the specific environments that bacterial species occupy [36]. It is plausible that emergence of connector-mediated regulation, which leads to persistence of expression of the polymyxin B resistance operon *pbgP* under the conditions of low Mg^2+^ (Figure 5B, D, F), contributed to the ability of *K. pneumoniae* and *S. enterica* to survive in soil environments [37],[38]. (Notably, *Y. pestis*, which lacks the connector protein PmrD, is reported to survive in soil only for short periods of time [39].) Indeed, polymyxin B is present in soil as a result of natural activity of some bacteria [40]. Additionally, the lipopolysaccharide (LPS) modifications brought about by the *pbgP* operon products confer resistance to metal ions such as Fe^3+^ and Al^3+^, which are abundant in soil [41]. This could explain the advantage of activating *pbgP* under high Fe^3+^ conditions sensed by the PmrA/PmrB system [20]. The benefit of *pbgP* induction by low Mg^2+^ (sensed by the PhoP/PhoQ system) may come from the fact that Mg^2+^ normally neutralizes the negative charges in the LPS [42]; thus, when the levels of Mg^2+^ are low, the LPS is chemically modified by PmrA-activated gene products that neutralize these negative charges [2]. It is likely that the rapid activation and delayed deactivation of *pbgP*, as well as the heightened *pbgP* expression level promoted by the FCL architecture (Figure 5E, F), contribute to the lifestyle of *K. pneumoniae*, including its ability to survive in soil for extended times [37].

Environmental selection of genetic regulatory circuits can be analyzed within the framework of cost–benefit theory [43],[44]. For example, it has been shown that the FFL with AND logic has a selective advantage over the direct regulation circuit (with an AND-gate) in environments where the duration distribution for an input pulse is sufficiently broad (both long and short pulses are probable) [43]. Because the FCL is expected to act as a strong sign-sensitive delay element (stronger than the FFL) (Figure 5A, B), it is conceivable that the FCL is the preferred design in environments where delayed activation and rapid deactivation result in a selective disadvantage.

## Materials and Methods

The bacterial strains, plasmids, growth conditions and construction of deletion mutants are described in Text S1. The list of strains and plasmids used in this study is given in Table S1.

### RNA Isolation

To isolate the RNA used in the S1 nuclease assay (Figure 4A), overnight cultures of *K. pneumoniae* grown in N-minimal medium containing 10 mM Mg^2+^ were washed and diluted 1∶50 into 50 ml of N-minimal medium containing either 10 µM MgCl_2_, 10 mM MgCl_2_ or 10 µM MgCl_2_ and 100 µM FeSO_4_. Total RNA was extracted from early-logarithmic phase cultures (OD_600_, 0.250) with the MasterPure RNA purification kit (Epicentre Technologies) according to the manufacturer's recommended protocol.

### S1 Nuclease Assay

Double stranded DNA probes to the *pbgP* promoter regions of *K. pneumoniae* were generated by PCR using the primers 3249 (5′-TTCGTGACAGGAACGCATCT′-3′) and 3250 (5′-GGGCGCGAAAAAGGCAAAAA-3′). S1 nuclease reactions were performed as described previously [12]. Hybridization products were analyzed by electrophoresis on a 6% polyacrylamide, 7.5 M urea gel and compared with Maxam-Gilbert A+G DNA ladders generated from the appropriate DNA probe. Assays were performed in triplicate.

### DNase I Footprinting

DNase I footprinting was performed as described previously [12]. The *K. pneumoniae pbgP* promoter region probe was generated as described in Materials and Methods. The *S. enterica* PhoP and PmrA proteins were purified as described previously [45]. Samples were analyzed by electrophoresis on a 6% polyacrylamide, 7.5 M urea gel and compared with a Maxam-Gilbert A+G DNA ladder generated from the same DNA probe.

### GFP Expression Assay

*K. pneumoniae* strains harboring the pAG, pAG-*rpsM*, pAG-*pmrD_Klebsiella_* plasmids were grown in N-minimal media, pH 7.7 or 5.8, containing 38 mM glycerol with either 10 µM MgCl_2_, 10 mM MgCl_2_ or 10 µM MgCl_2_ and 100 µM FeSO_4_ and supplemented with 10 µg/ml tetracycline. GFP expression was analyzed following 4 hours of growth at 37°C using a Becton Dickinson fluorescent-activated cell sorter. Assays were performed in triplicate. Error bars (Figure 3B) indicate standard deviation.

### Computational Sequence Analysis

Identification of protein orthologs and putative transcription factor binding sites is described in Text S1. For phylogenetic reconstruction, the amino acid sequences encoded by three housekeeping genes (*gapA*, *groEL* and *gyrA*) were concatenated to infer the molecular phylogeny for the eight enteric species [46] (Figure 2). Sequences were aligned using ClustalX and subjected to maximum parsimony and nonparametric bootstrap resampling analysis as implemented in PAUP* (version 4.0b10). The tree was rooted with *Pseudomonas aeruginosa* as the outgroup.

### Quantitative Analysis of Transcription

To test *pmrD* transcription (Figure S2), RNA was isolated from *K. pneumoniae* strains EG13127, EG13129 and EG15289, and the quantification of *pmrD* mRNA levels were performed as described [47] with the following modifications: aliquot of cells was taken at 1 hour post-induction, and the PCR analysis was performed using Fast SYBR Green Master Mix and a 7500 Fast Real-Time PCR System (Applied Biosystems, Foster City, CA). The following primers were used in the real-time PCR analysis (5′ to 3′): 7873 (TCTGCCGCGTCGTGC, *pmrD* forward), 7874 (CAATCTCTGCGATCATCTCCAG, *pmrD* reverse), 8813 (TTGACGTTACCCGCAGAAGAA, *rrs*, forward), 8816 (GCGCTTTACGCCCAGTAATT, *rrs*, reverse). Data were normalized with the values corresponding to 16S RNA, and represent five independent experiments with the highest and lowest outliers omitted. Error bars (Figure S2) correspond to standard deviation.

The activation and deactivation experiments (Figure 5E,F) with the *S. enterica* strains 14028s, EG17353 and EG17354, including *pbgP* mRNA isolation and quantification using real-time-PCR, were performed as described [2] with the following modifications: the reverse transcription reaction was run with ∼6.5 ng total RNA, and the PCR analysis was performed using a 7500 Fast Real-Time PCR System (Applied Biosystems, Foster City, CA). Activation time-course measurements done over larger time intervals have produced results similar to those shown in Figure 5E. In the PCR reaction, the following primers were used (5′ to 3′): 6522 (TGATGTCGGACTTTTTGCCTT, *pbgP*, forward), 6523 (GCTCTTCCGCGCCCAT, *pbgP*, reverse), 3023 (CCAGCAGCCGCGGTAAT, *rrs*, forward), 3024 (TTTACGCCCAGTAATTCCGATT, *rrs*, reverse). Data were normalized with the values corresponding to 16S RNA. Measurements were done in duplicate; error bars (Figure 5E, F) correspond to standard deviation.

### Mathematical Modeling

The mathematical models of the FFL, FCL and direct regulation circuit are systems of ordinary differential equations (ODEs) that describe concentration dynamics for the main chemical components of the three regulatory circuits. The FCL model comprises five ODEs for the PmrD, PmrA, PmrA-P, the PmrD/PmrA-P complex, and *pbgP* mRNA concentrations (Equations 1–5 in Text S1). The FFL and direct regulation models consist of three equations each; the equations describe changes in the concentrations of PmrA, PmrA-P, and *pbgP* mRNA (Equations 6–11 in Text S1). In all models, the concentration of PhoP-P is an external variable representing the main input; the chemical reactions are modeled by using mass action kinetics, and transcriptional control is described with sigmoidal functions [2],[48]. The activation dynamics of PhoP-P was modeled using piecewise Hermite interpolating polynomials fitted to the experimental data for PhoP-P activation dynamics [47]; deactivation dynamics was modeled with an exponential decay function (see Text S1). The balance of phosphorylation and dephosphorylation rates for PmrA (and for protein Y of the FFL, Figure 1D) represented the second input of the circuits; we consider the situations when this input is strongly activated (high phosphorylation rate) or mildly activated (low phosphorylation rate). For all computational experiments, the initial concentrations (at time 0) were the steady-state concentrations corresponding to the PhoP-P level at time 0.

All computations were performed in MATLAB R2007a (MathWorks, Natick, MA). In delay distribution computations, the delays were defined as the differences between the activation and deactivation times for the FCL (or FFL) and those for the direct regulation circuit. Activation time was defined as the time required to reach an activation level equal to *inactive level*+(*activatedlevel*−*inactive level*)/10; deactivation time was defined in an analogous way. Activation and deactivation delays correspond to situations when the PhoP-P input of the circuits was activated and deactivated, respectively.

The delay distributions for the FCL (Figure 5A, B, C, D) were simulated as follows: parameter values for the models in the simulations were sampled independently from uniform distributions over intervals provided in Table S2. While the real-life parameter value distributions for the genetic regulatory systems are unknown, in our choice of uniform distributions we followed the established methodology of statistical analysis for biochemical pathways [49]. A pair of randomly generated parameter sets, one for the FCL and the other one for the direct regulation circuit, was accepted or rejected depending on whether the model outputs for these models satisfied certain filtering criteria (Text S1). The purpose of filtering was to retain only the parameter sets that rendered functional regulatory circuits [3]. The pairs of parameter sets were generated randomly until the number of accepted pairs was equal to 1000. These parameter sets were used to calculate model trajectories necessary for the estimation of activation and deactivation delays of the FCL with respect to the direct regulation circuit. The delay distributions for the FFL (Figure 5A, B, C, D) were simulated in an analogous fashion. To test the robustness of the simulation results, we applied alternative sampling strategies (used to produce Figures S4–[Supplementary-material pgen.1000233.s005] S6), which, along with the details of our simulation procedures, are described in Text S1.

## Supporting Information

Figure S1 Alignment of the amino acid sequences for the PmrD proteins from *E. coli* K-12, *S. enterica* serovar Typhimurium strain LT2, and *K. pneumoniae* strain KC2668. The sequences were aligned using Clustal W 1.83.
(0.01 MB PDF)

Figure S2 Transcription from the *pmrD* promoter in *K. pneumoniae* is PhoP-dependent but PmrA-independent. mRNA levels of *pmrD* were measured by real-time PCR analysis using isolated RNA from wild-type (EG13127) and isogenic *phoP* (EG15289) and *pmrA* (EG13129) *K. pneumoniae* strains following growth in N-minimal medium, pH 7.7, containing 38 mM glycerol with 50 µM Mg^2+^ and 100 µM Fe^3+^ (see main text, Materials and Methods). The mRNA levels are normalized to 16S RNA.
(0.01 MB PDF)

Figure S3 DNase I footprinting analysis of the *K. pneumoniae pbgP* promoter performed for the non-coding strands. (A) Footprinting analysis of the *pbgP* promoter with increasing amounts of the PhoP protein (0, 25, 75, 125 pmol). (B) DNase footprinting analysis of the *pbgP* promoter with increasing amounts of the PmrA protein (0, 10, 20, 40 pmol). Solid vertical lines correspond to regions of the *pbgP* promoter protected by the PhoP and PmrA proteins. The start and end positions of the protected regions are given relative to the transcription start site immediately downstream of the protected region (see Figure 4B). The affinity of the PhoP and PmrA proteins for the −10 to −44 and −19 to −44 regions is less than that corresponding to the −46 to −91 and −22 to −91 regions, respectively. This could be due to the presence of PhoP and PmrA half-boxes in at the ORF-proximal sites as opposed to complete boxes at the ORF-distal sites.
(0.33 MB PDF)

Figure S4 Delay length distributions for the feedforward connector loop (FCL) and feedforward loop (FFL). Activation and deactivation delays correspond to the situations when the PhoP-P input of the circuits was activated and deactivated, respectively. The delays are defined as differences between the activation and deactivation times for the FCL (or FFL) and those for the direct regulation circuit (Figure 1B). The distributions were estimated from simulations with mathematical models as described in Materials and Methods. In the simulations, the parameter values for the models were sampled using the small-noise strategy with noise level 0 (see Text S1). The second input with strong and mild activation corresponds to high and low phosphorylation/dephosphorylation rate ratio for PmrA (or for protein Y of the FFL), respectively. (A) Activation delay length distributions for the FCL and FFL. (B) Deactivation delay length distributions for the FCL and FFL. (C) Activation delay length distributions for the FCL and FFL lacking direct activation branches. (D) Deactivation delay length distributions for the FCL and FFL lacking direct activation branches.
(0.01 MB PDF)

Figure S5 Delay length distributions for the feedforward connector loop (FCL) and feedforward loop (FFL). Activation and deactivation delays correspond to the situations when the PhoP-P input of the circuits was activated and deactivated, respectively. The delays are defined as differences between the activation and deactivation times for the FCL (or FFL) and those for the direct regulation circuit (Figure 1B). The distributions were estimated from simulations with mathematical models as described in Materials and Methods. In the simulations, the parameter values for the models were sampled using the small-noise strategy with noise level 0.3 (see Text S1). The second input with strong and mild activation corresponds to high and low phosphorylation/dephosphorylation rate ratio for PmrA (or for protein Y of the FFL), respectively. (A) Activation delay length distributions for the FCL and FFL. (B) Deactivation delay length distributions for the FCL and FFL. (C) Activation delay length distributions for the FCL and FFL lacking direct activation branches. (D) Deactivation delay length distributions for the FCL and FFL lacking direct activation branches.
(0.01 MB PDF)

Figure S6 Delay length distributions for the feedforward connector loop (FCL) and feedforward loop (FFL). Activation and deactivation delays correspond to the situations when the PhoP-P input of the circuits was activated and deactivated, respectively. The delays are defined as differences between the activation and deactivation times for the FCL (or FFL) and those for the direct regulation circuit (Figure 1B). The distributions were estimated from simulations with mathematical models as described in Materials and Methods. In the simulations, the parameter values for the models were sampled using the small-noise strategy with noise level 0.95 (see Text S1). The second input with strong and mild activation corresponds to high and low phosphorylation/dephosphorylation rate ratio for PmrA (or for protein Y of the FFL), respectively. (A) Activation delay length distributions for the FCL and FFL. (B) Deactivation delay length distributions for the FCL and FFL. (C) Activation delay length distributions for the FCL and FFL lacking direct activation branches. (D) Deactivation delay length distributions for the FCL and FFL lacking direct activation branches.
(0.01 MB PDF)

Table S1 List of strains and plasmids used in this study.
(0.14 MB PDF)

Table S2 Intervals used for model parameter sampling. The superscripts for the parameters are omitted, because the same intervals are used to sample the corresponding parameters for the FCL, FFL, and the direct regulation circuit.
(0.06 MB PDF)

Text S1 Supplementary methods.
(0.08 MB PDF)
