# Supplementary material for: Evolution and Dynamics of Regulatory Architectures Controlling Polymyxin B Resistance in Enteric Bacteria
Source: PLoS Genet. 2008 Oct 24;4(10):e1000233. doi: 10.1371/journal.pgen.1000233 (PMC2565834; doi:10.1371/journal.pgen.1000233)
Supplement: Figure S1 — Alignment of the amino acid sequences for the PmrD proteins from E. coli K-12, S. enterica serovar Typhimurium strain LT2, and K. pneumoniae strain KC2668. The sequences were aligned using Clustal W 1.83. (0.01 MB PDF) [file pgen.1000233.s001.pdf]

```
E. coli           MEWLVKKSCCNKQDNRHVLMLCDAGGAIKMIAEVKS-DFAVKVGDLLSPLQNALYCINRE
S. enterica       MEWLVKKSHYVKKRACHVLVLCDSGGSLKMIAEANS-MILLSPGDILSPLQDAQYCINRE
K. pneumoniae     MEWWVKKVQDNASASLCRVVLQS--GALEMIAEIEACRLRLREGDKLTPLADARYCLNNN
                  *** ***     .     ::* .  *:::**** ::  : :  ** *:** :* **:*.:

E. coli           KLHTVKVLSASSYSPDEWERQCKVAGKTQ 88
S. enterica       KHQTLKIVDARCYSCDEWQRLTRKPS--- 85
K. pneumoniae     PTQTLKIRNATHYSSERWTNAGK------ 81
                    :*:*: .*  ** :.* .  :
```
