# Supplementary figures and images for: Evolution and Dynamics of Regulatory Architectures Controlling Polymyxin B Resistance in Enteric Bacteria
Source: PLoS Genet. 2008 Oct 24;4(10):e1000233. doi: 10.1371/journal.pgen.1000233 (PMC2565834; doi:10.1371/journal.pgen.1000233)

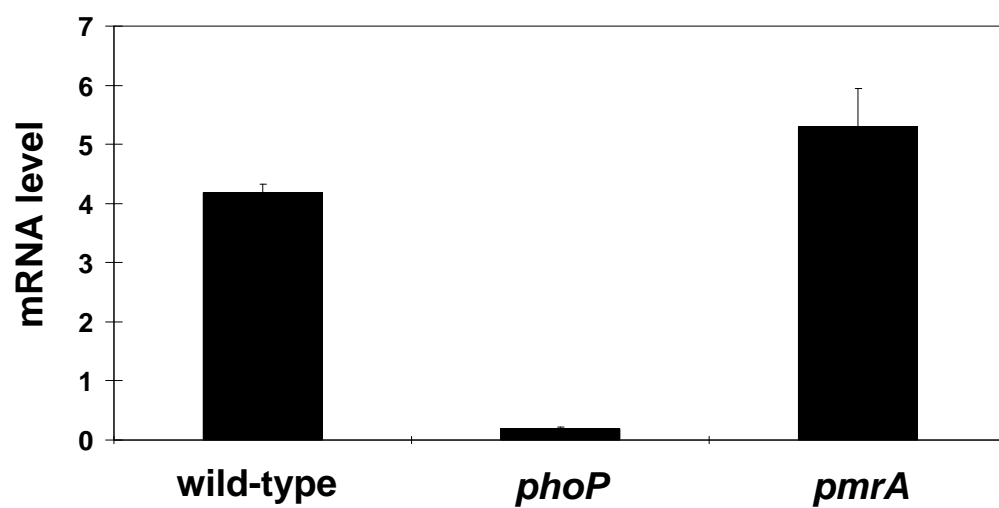

Supplement: Figure S2 — Transcription from the pmrD promoter in K. pneumoniae is PhoP-dependent but PmrA-independent. mRNA levels of pmrD were measured by real-time PCR analysis using isolated RNA from wild-type (EG13127) and isogenic phoP (EG15289) and pmrA (EG13129) K. pneumoniae strains following growth in N-minimal medium, pH 7.7, containing 38 mM glycerol with 50 µM Mg2+ and 100 µM Fe3+ (see main text, Materials and Methods). The mRNA levels are normalized to 16S RNA. (0.01 MB PDF) [file pgen.1000233.s002.pdf]

**A**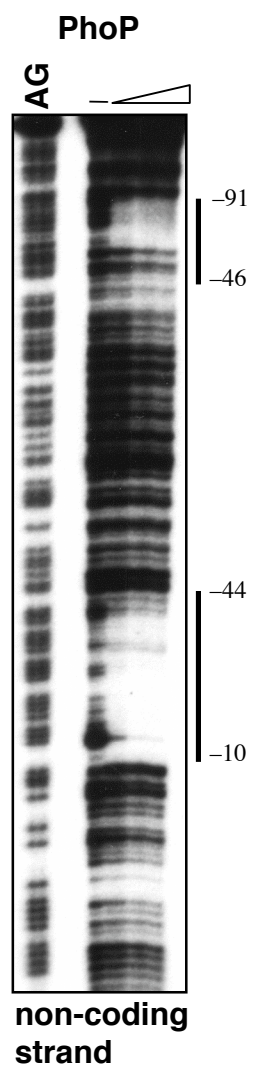**B**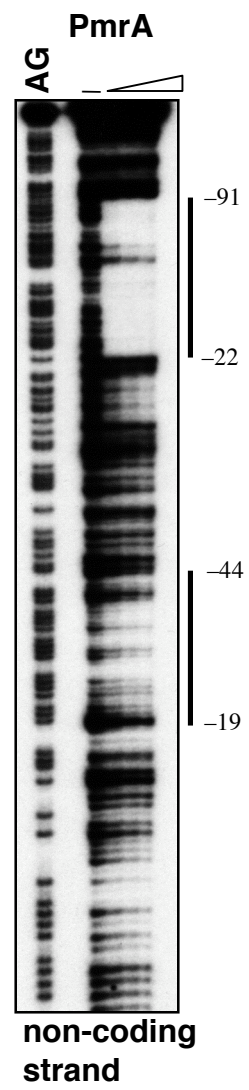

Supplement: Figure S3 — DNase I footprinting analysis of the K. pneumoniae pbgP promoter performed for the non-coding strands. (A) Footprinting analysis of the pbgP promoter with increasing amounts of the PhoP protein (0, 25, 75, 125 pmol). (B) DNase footprinting analysis of the pbgP promoter with increasing amounts of the PmrA protein (0, 10, 20, 40 pmol). Solid vertical lines correspond to regions of the pbgP promoter protected by the PhoP and PmrA proteins. The start and end positions of the protected regions are given relative to the transcription start site immediately downstream of the protected region (see Figure 4B). The affinity of the PhoP and PmrA proteins for the −10 to −44 and −19 to −44 regions is less than that corresponding to the −46 to −91 and −22 to −91 regions, respectively. This could be due to the presence of PhoP and PmrA half-boxes in at the ORF-proximal sites as opposed to complete boxes at the ORF-distal sites. (0.33 MB PDF) [file pgen.1000233.s003.pdf]

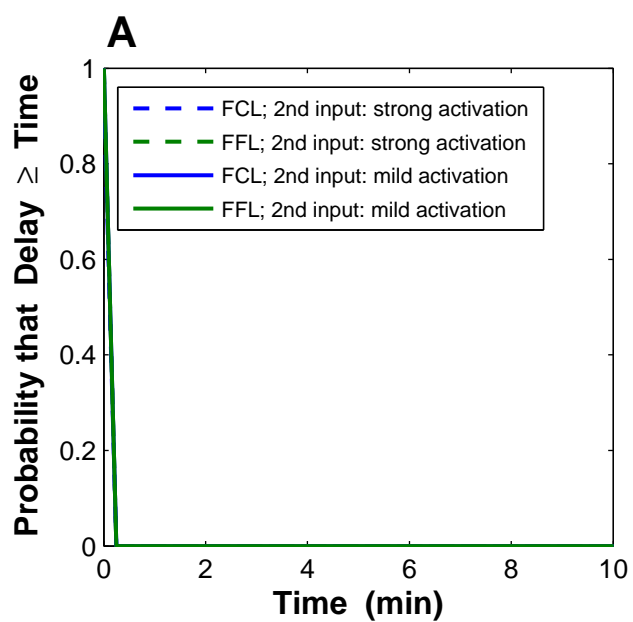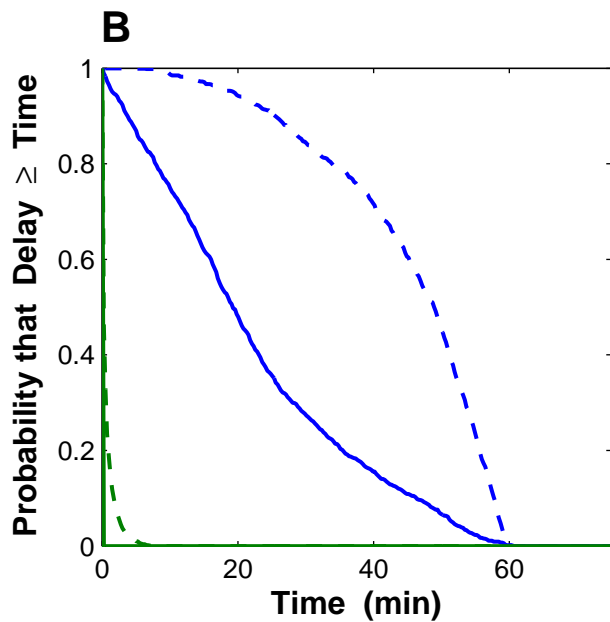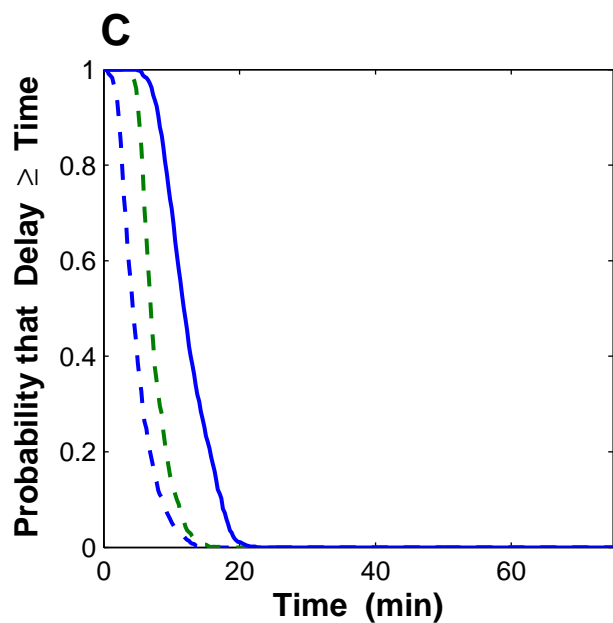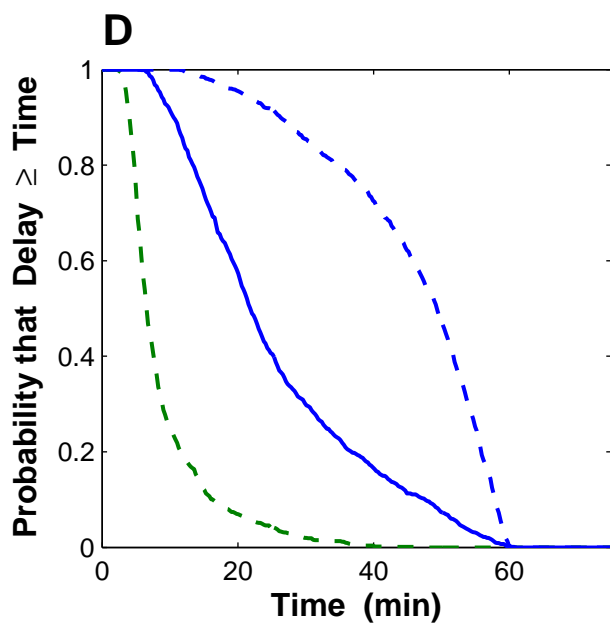

Supplement: Figure S4 — Delay length distributions for the feedforward connector loop (FCL) and feedforward loop (FFL). Activation and deactivation delays correspond to the situations when the PhoP-P input of the circuits was activated and deactivated, respectively. The delays are defined as differences between the activation and deactivation times for the FCL (or FFL) and those for the direct regulation circuit (Figure 1B). The distributions were estimated from simulations with mathematical models as described in Materials and Methods. In the simulations, the parameter values for the models were sampled using the small-noise strategy with noise level 0 (see Text S1). The second input with strong and mild activation corresponds to high and low phosphorylation/dephosphorylation rate ratio for PmrA (or for protein Y of the FFL), respectively. (A) Activation delay length distributions for the FCL and FFL. (B) Deactivation delay length distributions for the FCL and FFL. (C) Activation delay length distributions for the FCL and FFL lacking direct activation branches. (D) Deactivation delay length distributions for the FCL and FFL lacking direct activation branches. (0.01 MB PDF) [file pgen.1000233.s004.pdf]

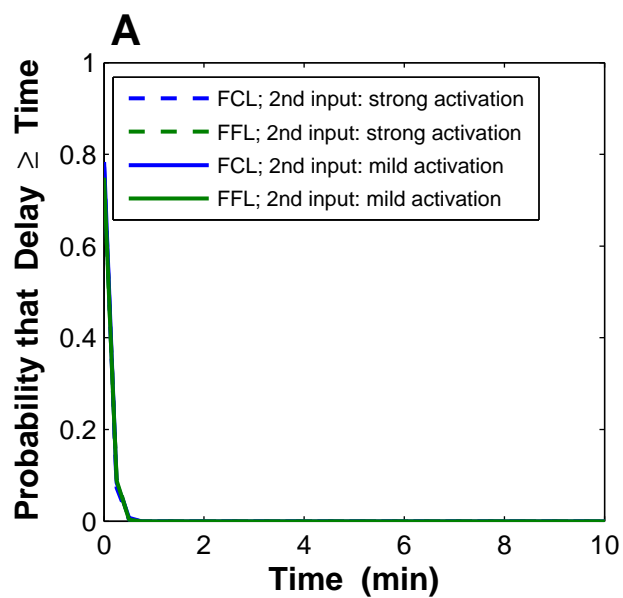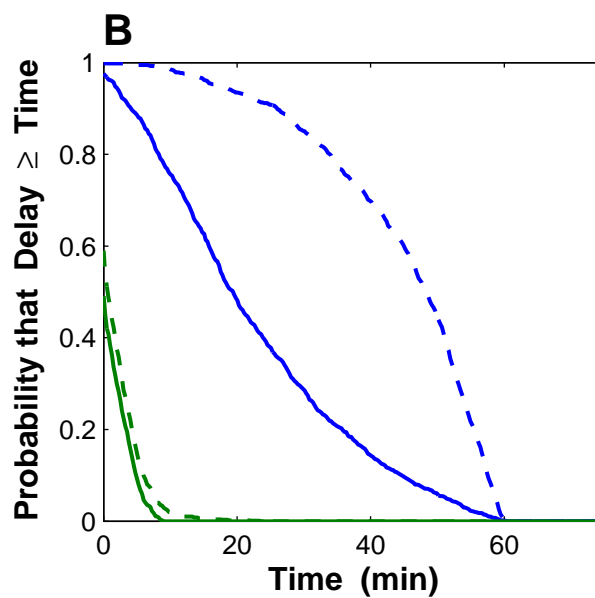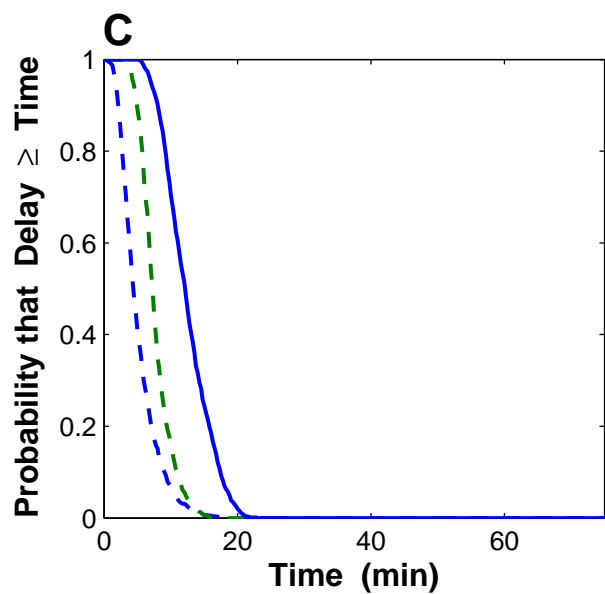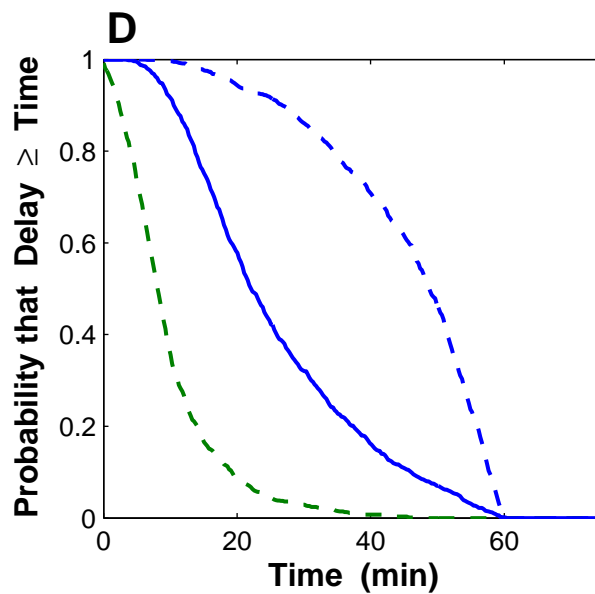

Supplement: Figure S5 — Delay length distributions for the feedforward connector loop (FCL) and feedforward loop (FFL). Activation and deactivation delays correspond to the situations when the PhoP-P input of the circuits was activated and deactivated, respectively. The delays are defined as differences between the activation and deactivation times for the FCL (or FFL) and those for the direct regulation circuit (Figure 1B). The distributions were estimated from simulations with mathematical models as described in Materials and Methods. In the simulations, the parameter values for the models were sampled using the small-noise strategy with noise level 0.3 (see Text S1). The second input with strong and mild activation corresponds to high and low phosphorylation/dephosphorylation rate ratio for PmrA (or for protein Y of the FFL), respectively. (A) Activation delay length distributions for the FCL and FFL. (B) Deactivation delay length distributions for the FCL and FFL. (C) Activation delay length distributions for the FCL and FFL lacking direct activation branches. (D) Deactivation delay length distributions for the FCL and FFL lacking direct activation branches. (0.01 MB PDF) [file pgen.1000233.s005.pdf]

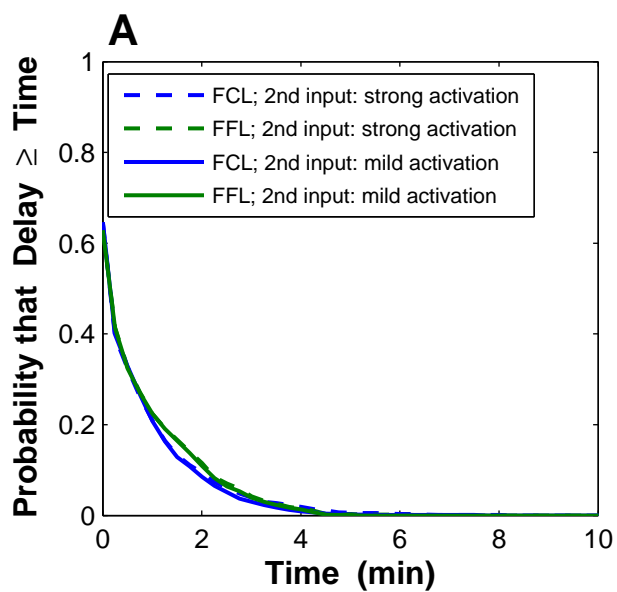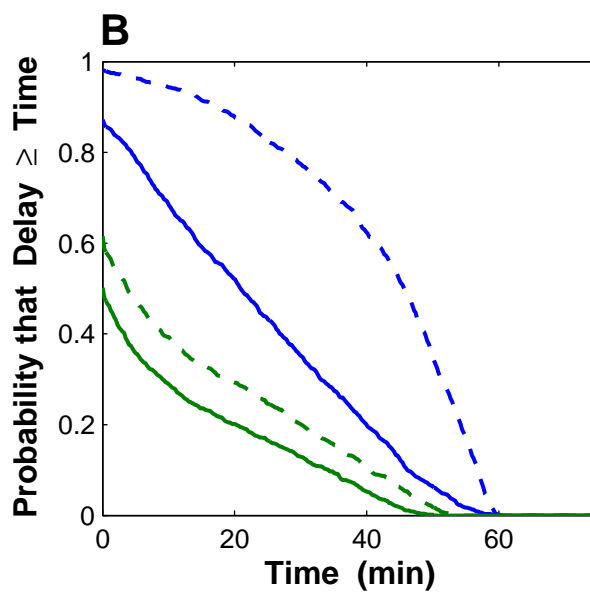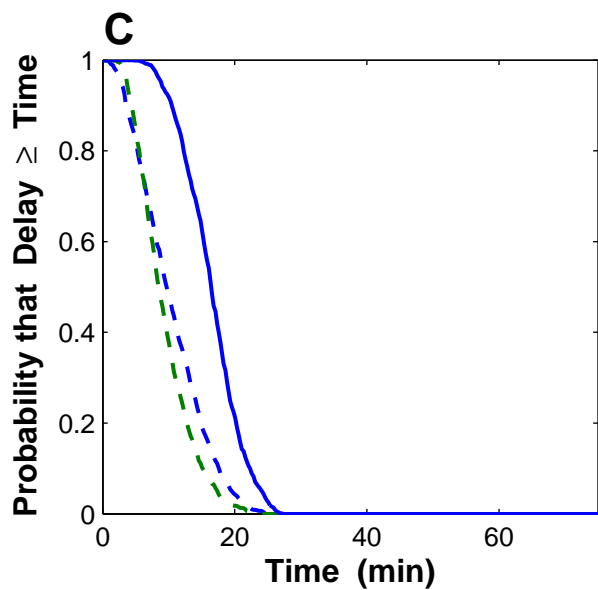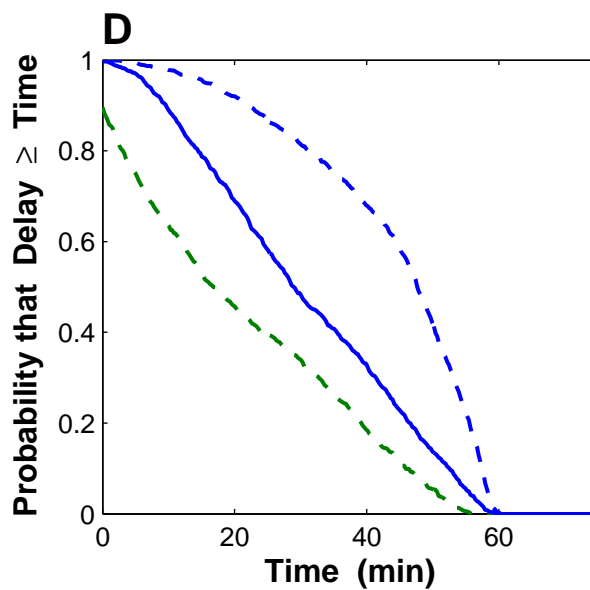

Supplement: Figure S6 — Delay length distributions for the feedforward connector loop (FCL) and feedforward loop (FFL). Activation and deactivation delays correspond to the situations when the PhoP-P input of the circuits was activated and deactivated, respectively. The delays are defined as differences between the activation and deactivation times for the FCL (or FFL) and those for the direct regulation circuit (Figure 1B). The distributions were estimated from simulations with mathematical models as described in Materials and Methods. In the simulations, the parameter values for the models were sampled using the small-noise strategy with noise level 0.95 (see Text S1). The second input with strong and mild activation corresponds to high and low phosphorylation/dephosphorylation rate ratio for PmrA (or for protein Y of the FFL), respectively. (A) Activation delay length distributions for the FCL and FFL. (B) Deactivation delay length distributions for the FCL and FFL. (C) Activation delay length distributions for the FCL and FFL lacking direct activation branches. (D) Deactivation delay length distributions for the FCL and FFL lacking direct activation branches. (0.01 MB PDF) [file pgen.1000233.s006.pdf]
