# Supplementary material for: Evolution and Dynamics of Regulatory Architectures Controlling Polymyxin B Resistance in Enteric Bacteria
Source: PLoS Genet. 2008 Oct 24;4(10):e1000233. doi: 10.1371/journal.pgen.1000233 (PMC2565834; doi:10.1371/journal.pgen.1000233)
Supplement: Table S1 — List of strains and plasmids used in this study. (0.14 MB PDF) [file pgen.1000233.s007.pdf]

Strain or Plasmid	Description	Reference*
Klebsiella pneumoniae		
EG13127	Δ(*bla*)-2 KC2668	Ref. ([1])
EG14740	Δ(*bla*)-2 Δ*pmrD*::Kn^R	This work
EG13129	Δ(*bla*)-2 Δ*pmrA*::Kn^R	Ref. ([25])
EG15289	Δ(*bla*)-2 Δ*phoP*::Kn^R	This work
EG14735	Δ(*bla*)-2 Δ*pbgP*::Kn^R	This work
EG16126	Δ(*bla*)-2 Kn^R-*pbgP*$^+$	This work
Salmonella enterica		
**serovar Typhimurium**		
14028s	Wild-type	Ref. ([26])
EG17353	$p_{pbgP\ Yersinia}$	This work
EG17354	$p_{pbgP\ Yersinia}$ *pmrD*1::Cm	This work
EG17235	p_{pbgP}::*tetRA*	Ref. ([5])
EG17343	p_{pbgP}::*tetRA* *pmrD*1::Cm	This work
EG11491	*pmrD*1::Cat	Ref. ([6])
**Plasmids**		
pAG	rep_{p15A} Tet^R *gfp*mut3a	Ref. ([7])
pAG-$pmrD_{Klebsiella}$	rep_{p15A} Tet^R *gfp*mut3a $PpmrD_{Klebsiella}$	This work

pAG-*rpsM*	rep_{p15A} Tet^{R} *gfp*mut3a P*rpsM*	Ref. ([7])
pKD3	$rep_{R6K\gamma}$ Ap^{R} FRT Cm^{R} FRT	Ref. ([3])
pKD4	$rep_{R6K\gamma}$ Ap^{R} FRT Kn^{R} FRT	Ref. ([3])
pKD46	$rep(ts)_{pSC101}$ Ap^{R} p_{araBAD} γ β exo^{+}	Ref. ([3])

*The references are provided in Text S1
