# Supplementary material for: Evolution and Dynamics of Regulatory Architectures Controlling Polymyxin B Resistance in Enteric Bacteria
Source: PLoS Genet. 2008 Oct 24;4(10):e1000233. doi: 10.1371/journal.pgen.1000233 (PMC2565834; doi:10.1371/journal.pgen.1000233)
Supplement: Table S2 — Intervals used for model parameter sampling. The superscripts for the parameters are omitted, because the same intervals are used to sample the corresponding parameters for the FCL, FFL, and the direct regulation circuit. (0.06 MB PDF) [file pgen.1000233.s008.pdf]

Parameter	Sampling interval
k_{pbgP}, $\mu M / \min$	(0.01, 0.1)
k_{-pbgP}, $\min^{-1}$	(0.09, 0.2)
k_{PmrD}, $\mu M / \min$	(1, 50)
k_{-PmrD}, $\min^{-1}$	(0.01, 0.2)
k_c, $1/(\min \cdot \mu M)$	(1, 10)
k_{-c}, $\min^{-1}$	(0.1, 1)
k_{PmrA}, $\mu M / \min$	(0.01, 0.1)
k_{-PmrA}, $\min^{-1}$	(0.01, 0.2)
$k_p^{\max}$, $\min^{-1}$	(0.5, 5)
$k_{-p}^{\max}$, $\min^{-1}$	(1, 10)
K_1, $(\mu M)^{-2}$	(1, 30)
K_2, $(\mu M)^{-2}$	(1, 30)
K_3, $(\mu M)^{-2}$	(1, 30)
