# Supplementary material for: Evolution and Dynamics of Regulatory Architectures Controlling Polymyxin B Resistance in Enteric Bacteria
Source: PLoS Genet. 2008 Oct 24;4(10):e1000233. doi: 10.1371/journal.pgen.1000233 (PMC2565834; doi:10.1371/journal.pgen.1000233)
Supplement: Text S1 — Supplementary methods. (0.08 MB PDF) [file pgen.1000233.s009.pdf]

Text S1

Supplementary methods

Bacterial strains and growth conditions. Bacterial strains used in this study are listed in Table S1. *Klebsiella pneumoniae* strains were derived from Δ(*bla*)-2 KC2668, as reported [1]. *K. pneumoniae* and *Escherichia coli* strains were grown at 37 °C in N-minimal medium, pH 7.7 (as reported [2]), supplemented with 0.1% casamino acids, 38 mM glycerol and 10 µM $MgCl_2$ (20 µM $MgCl_2$ for *Salmonella enterica*), 10 mM $MgCl_2$ or 10 µM $MgCl_2$ and 100 µM $FeSO_4$ as indicated. *E. coli* DH5α was used as the host for preparation of plasmid DNA. Ampicillin and kanamycin were used at 50 µg/ml, chloramphenicol at 20 µg/ml and tetracycline at 10 µg/ml.

Construction of *K. pneumoniae* chromosomal mutants. All mutant strains were constructed according to the one-step disruption protocol [3] using the following primer pairs. *K. pneumoniae pmrD*, 3639 (5'-ATTATCGACGCACATGTCGCTATGAGAGGATAAAAAACAGCATATGAA TATCCTCCTTAG-3') and 3640 (5'-CGATACTCAGACCATAACCAGTAAAAGG AGCGGGCTCGTGGTGTAGGCTGGAGCTGCTTC-3'), *K. pneumoniae phoP*, 4335 (5'-GAGCTTCAGACTACTATCGAAAATGCTAGGGAGAACAGCCGTGTAGGCTGGAGCTG CTTC-3') and 4336 (5'-CCCGTAATGACAGCGGGAAGATATGCCGCAACAGT CCCTTCATATGAATATCCTCCTTAG-3'), *K. pneumoniae pbgP*, 4333 (5'-CGACCCA GATTCGTGCGTGGGTCATCTACAGAACAGGATGGTGTAGGCTGGAGCTGCTTC-3') and 4334 (5'-AACCACCGAGACCTTCTTCACGGGAGGGTAAGTAAGCATACA TATGAATATCCTCCTTAG-3'). Mutant alleles were verified by PCR [3] and Southern hybridization (data not shown).

Construction of *S. enterica* chromosomal mutants. Strain EG17353, which has the *Y. pestis* *pbgP* promoter instead of the original *S. enterica pbgP* promoter, was constructed by a combination of the one-step gene inactivation method and the counter selection method for Tet^s colonies [4]. A PCR fragment containing the *Y. pestis pbgP* promoter was amplified using primers 6109 and 6110 and *Y. pestis* KIM6 chromosomal DNA as template and recombined into the chromosome of strain EG17235 [5], replacing the intergenic region between the *pmrG* and *pbgP* genes including the *tetRA* insertion. Strain EG17354 was constructed by phage P22-mediated transduction of strain EG17353 with a P22 lysate prepared on the *pmrD1*::Cm strain EG17343, which harbors the *pmrD1*::Cm allele from strain EG11491[6], selecting for chloramphenicol-resistant transductants.

Plasmid construction. Plasmid pAG-$pmrD_{Klebsiella}$ was constructed by cloning between the EcoRI and KpnI sites of the pAG vector [7] a PCR fragment containing the *pmrD* promoter region from *K. pneumoniae* generated with primers 4420 (5'- CGGAATTCGCT TCATGACGCTCTCTCTT -3') and 4421 (5'- GGGGTACCGCCGAAGCGTTGTCCTGTAC - 3') and digested with EcoRI and KpnI.

Identification of protein orthologs and putative transcription factor binding sites. The homologs to the *S. enterica* PhoP, PmrA, PmrD, and PbgP proteins were either identified or confirmed based on tBLASTn searches conducted directly against the following genomes *Erwinia carotovora* subsp. *atroseptica* [8], *E. coli* K-12 strain MG1655 [9], *K. pneumoniae* (http://www.genome.wustl.edu), *Photorhabdus luminescens* subsp. *laumondii* TT01 [10], *Shigella flexneri* 2457T [11], *Serratia marcescens* (http://www.sanger.ac.uk), and *Yersinia pestis* KIM [12]. Orthology of the PhoP and PmrA proteins was assigned based on phylogenetic analysis. PhoP, PmrA, as well as the sequences for the closely related OmpR, YgiX and TctD re-

sponse regulator protein were aligned using ClustalX [13] and subjected to maximum parsimony and nonparametric bootstrap resampling analysis as implemented in PAUP* (version 4.0b10). Orthology of the PbgP proteins was assigned based on genomic context. All *pbgP* homologues were found to be the first gene in a seven-gene operon [14]. Promoter region of the *pbgP* orthologs were examined manually and with SOAR-TOOLS Binding Site Searching (http://soar-tools.wustl.edu) for the consensus binding sites for the PhoP [15-17] and PmrA [18-20] proteins.

Description of the mathematical models

The mathematical models for the feedforward connector loop (FCL), the feedforward loop (FFL), and the direct regulation pathway describe the changes in the concentration of *pbgP* mRNA resulting from the changes in the input variable, [PhoP-P]. The ODEs (ordinary differential equations) constituting the FCL model are given below (the gray boxes under each equation contain the corresponding chemical reactions):

$$\frac{dX_1}{dt} = k_{pbgP}^{fcl}\left(1 - \frac{1}{(1+K_2^{fcl}[\text{PhoP - P}]^2)(1+K_3^{fcl}(X_4^2+X_5^2))}\right) - k_{-pbgP}^{fcl}X_1; \quad (1)$$

$$\textit{pbgP}\ \text{promoter} \xrightarrow{k_{pbgP}^{fcl}(1-\cdots)} \textit{pbgP}\ \text{mRNA}\,(X_1) \xrightarrow{k_{-pbgP}^{fcl}} 0\ (\text{degradation} + \text{dilution})$$

$$\frac{dX_2}{dt} = k_{PmrD}^{fcl}\frac{K_1^{fcl}[\text{PhoP - P}]^2}{1+K_1^{fcl}[\text{PhoP - P}]^2} - k_{-PmrD}^{fcl}X_2 - k_c^{fcl}X_2X_4 + k_{-c}^{fcl}X_5; \quad (2)$$

$$\textit{pmrD}\ \text{promoter} \xrightarrow{k_{PmrD}^{fcl}\frac{K_1^{fcl}[\text{PhoP-P}]^2}{1+K_1^{fcl}[\text{PhoP-P}]^2}} \text{PmrD}\,(X_2) \xrightarrow{k_{-PmrD}^{fcl}} 0\ (\text{degradation} + \text{dilution})$$

$$\text{PmrD}(X_2) + \text{PmrA - P}\,(X_4) \xleftrightarrow{k_c^{fcl}/k_{-c}^{fcl}} \text{PmrD/PmrA - P}\,(X_5)$$

$$\frac{dX_3}{dt} = k_{PmrA}^{fcl} - k_{-PmrA}^{fcl}X_3 + k_{-p}^{fcl}X_4 - k_p^{fcl}X_3; \quad (3)$$

$$\textit{pmrA}\ \text{promoter} \xrightarrow{k_{PmrA}^{fcl}} \text{PmrA}\ (X_3) \xrightarrow{k_{-PmrA}^{fcl}} 0\ (\text{degradation} + \text{dilution})$$
$$\text{PmrA}\ (X_3) \xleftrightarrow{k_p^{fcl} / k_{-p}^{fcl}} \text{PmrA - P}\ (X_4)$$

$$\frac{dX_4}{dt} = k_p^{fcl} X_3 - k_{-p}^{fcl} X_4 + k_{-c}^{fcl} X_5 - k_c^{fcl} X_2 X_4; \tag{4}$$

$$\text{PmrA}\ (X_3) \xleftrightarrow{k_p^{fcl} / k_{-p}^{fcl}} \text{PmrA - P}\ (X_4)$$
$$\text{PmrD}\ (X_2) + \text{PmrA - P}\ (X_4) \xleftrightarrow{k_c^{fcl} / k_{-c}^{fcl}} \text{PmrD / PmrA - P}\ (X_5)$$

$$\frac{dX_5}{dt} = k_c^{fcl} X_2 X_4 - k_{-c}^{fcl} X_5 . \tag{5}$$

$$\text{PmrD}\ (X_2) + \text{PmrA - P}\ (X_4) \xleftrightarrow{k_c^{fcl} / k_{-c}^{fcl}} \text{PmrD / PmrA - P}\ (X_5)$$

For the FCL model, the correspondence between the concentration variables X_i and the chemical species is as follows: X_1 ~ *pbgP* mRNA; X_2 ~ the PmrD protein; X_3 ~ the PmrA protein; X_4 ~ phosphorylated PmrA (PmrA-P); X_5 ~ PmrA-P/PmrD (protein complex). The concentration of phosphorylated PhoP, $[\text{PhoP - P}]$, is a function of time, and the main input for our model. The concentration of the *pbgP* mRNA is the model's output. In Equations 1–5, k_{pbgP}^{fcl} is the transcription rate for the *pbgP* promoter (under full induction conditions); k_{PmrD}^{fcl} and k_{PmrA}^{fcl} are the synthesis rates for the PmrD and PmrA proteins, respectively. The quantities k_{-pbgP}^{fcl}, k_{-PmrD}^{fcl}, and k_{-PmrA}^{fcl} are the degradation/dilution rates for the *pbgP* mRNA, PmrD, and PmrA, respectively. The constants k_p^{fcl} and k_{-p}^{fcl} are the respective rates of PmrA phosphorylation and dephosphorylation; k_c^{fcl} and k_{-c}^{fcl} are the rates of formation and decay of the PmrA-P/PmrD complex. Finally, K_1^{fcl} and K_2^{fcl} are the affinities for the interaction of PhoP-P with the *pmrD*

and *pbgP* promoters, respectively; K_3^{fcl} is the affinity for the interaction of PmrA-P with the *pbgP* promoter.

For the FFL model, the constituting ODEs are as follows:

$$\frac{dX_1}{dt} = k_{pbgP}^{ffl}\left(1 - \frac{1}{(1+K_2^{ffl}[\text{PhoP-P}]^2)(1+K_3^{ffl}X_3^2)}\right) - k_{-pbgP}^{ffl}X_1; \tag{6}$$

$$pbgP\ \text{promoter} \xrightarrow{k_{pbgP}^{ffl}(1-\cdots)} pbgP\ \text{mRNA}\,(X_1) \xrightarrow{k_{-pbgP}^{ffl}} 0\ (\text{degradation}+\text{dilution})$$

$$\frac{dX_2}{dt} = k_{PmrA}^{ffl}\frac{K_1^{ffl}[\text{PhoP-P}]^2}{1+K_1^{ffl}[\text{PhoP-P}]^2} - k_{-PmrA}^{ffl}X_2 + k_{-p}^{ffl}X_3 - k_p^{ffl}X_2; \tag{7}$$

$$pmrA\ \text{promoter} \xrightarrow{k_{PmrA}^{ffl}\frac{K_1^{ffl}[\text{PhoP-P}]^2}{1+K_1^{ffl}[\text{PhoP-P}]^2}} \text{PmrA}\ (X_2) \xrightarrow{k_{-PmrA}^{ffl}} 0\ (\text{degradation}+\text{dilution})$$

$$\text{PmrA}\ (X_2) \xleftrightarrow{k_p^{ffl}/k_{-p}^{ffl}} \text{PmrA-P}\ (X_3)$$

$$\frac{dX_3}{dt} = k_p^{ffl}X_2 - k_{-p}^{ffl}X_3\,. \tag{8}$$

$$\text{PmrA}\ (X_2) \xleftrightarrow{k_p^{ffl}/k_{-p}^{ffl}} \text{PmrA-P}\ (X_3)$$

In Equations 6–8, the correspondence between the concentration variables X_i and the chemical species is as follows: X_1 ~ *pbgP* mRNA; X_2 ~ the PmrA protein; X_3 ~ phosphorylated PmrA (PmrA-P) (here, *pbgP* corresponds to gene *z*, and PhoP and PmrA correspond to proteins X and Y, respectively – see main text, Figure 1D). The meaning of the rate and equilibrium constants is analogous to that of the corresponding parameters in Equations 1–5 (the corresponding parameters are the ones with identical subscripts).

For the direct regulation model, the constituting ODEs are as follows:

$$\frac{dX_1}{dt} = k_{pbgP}^{dir}\left(1 - \frac{1}{(1+K_2^{dir}[\text{PhoP-P}]^2)(1+K_3^{dir}X_3^2)}\right) - k_{-pbgP}^{dir}X_1; \tag{9}$$

$$pbgP\ \text{promoter} \xrightarrow{k_{pbgP}^{dir}(1-\cdots)} pbgP\ \text{mRNA}\,(X_1) \xrightarrow{k_{-pbgP}^{dir}} 0\ (\text{degradation} + \text{dilution})$$

$$\frac{dX_2}{dt} = k_{PmrA}^{dir} - k_{-PmrA}^{dir} X_2 + k_{-p}^{dir} X_3 - k_p^{dir} X_2; \tag{10}$$

$$pmrA\ \text{promoter} \xrightarrow{k_{PmrA}^{dir}} \text{PmrA}\,(X_2) \xrightarrow{k_{-PmrA}^{dir}} 0\ (\text{degradation} + \text{dilution})$$
$$\text{PmrA}\,(X_2) \xleftrightarrow{k_p^{dir}/k_{-p}^{dir}} \text{PmrA-P}\,(X_3)$$

$$\frac{dX_3}{dt} = k_p^{dir} X_2 - k_{-p}^{dir} X_3. \tag{11}$$

$$\text{PmrA}\,(X_2) \xleftrightarrow{k_p^{dir}/k_{-p}^{dir}} \text{PmrA-P}\,(X_3)$$

In Equations 9–11, the correspondence between the concentration variables X_i and the chemical species is as follows: X_1 ~ *pbgP* mRNA; X_2 ~ the PmrA protein; X_3 ~ phosphorylated PmrA (PmrA-P). The meaning of the rate and equilibrium constants is analogous to that for the corresponding parameters in Equations 1–5.

As mentioned in the main text (Materials and Methods), the initial conditions for Equations 1–11 are the solutions of the corresponding steady-state equations for a given PhoP-P concentration. The steady-state equations can be obtained from Equations 1–11 by setting the derivatives on the left-hand sides equal to 0. These equations are straightforwardly solved analytically; the solutions are unique.

Derivation details for the mathematical models

We rely on the model-building methodology described earlier [5]; below we mention some notable details. We extend the previously published [5] approach to account for possible variations in the PmrA levels, because in the FFL (Equations 6–8) the regulation of PmrA by PhoP occurs at the transcriptional level. Thus, changes in the PmrA concentration are essential for the indi-

rect control of *pbgP* expression by PhoP. Fitting the connector-mediated pathway model to experimental data showed that the exponential depletion rate of PmrD/PmrA-P due to degradation and dilution is small compared to other parameter values [5]; thus, in Equation 5, we neglect the corresponding term. We also used this result to neglect the degradation+dilution term for PmrA-P in Equation 4.

In our models (Equations 1–11), the *pbgP* operon can be activated by PhoP-P and PmrA-P, which non-competitively bind to the promoter (we assume that PmrA-P/PmrD has the same binding properties as PmrA-P). For this type of control, the mRNA production rate can be expressed as kp, where k is the maximum possible synthesis rate, and p is the probability that the promoter is bound by at least one activator [5,21]. In the case of the FCL,

$$p = 1 - \frac{1}{(1 + K_2^{fcl}[\text{PhoP - P}]^2)(1 + K_3^{fcl}(X_4^2 + X_5^2))}$$

(see Equation 1). To derive this expression, we combined the general strategy for modeling transcriptional regulation by two proteins [21] and the previously developed approaches to modeling *pbgP* regulation [5]. The expressions for p in the case of the FFL (Equation 6) and direct regulation (Equation 9) are similar.

The concentration of PhoP-P determines the main input of our transcription regulation models. The activation dynamics of PhoP-P is represented by the interpolation of the experimental PhoP-P dynamics data [22] via MATLAB's function pchip (piecewise cubic Hermite interpolating polynomial). Deactivation dynamics was modeled using the function

$$c(t) = (c_0 - c_{\lim})e^{-rt} + c_{\lim},$$

where $c_0 = 2$ μM is the initial concentration of PhoP-P, $c_{\lim} = 0.02$ μM is the concentration of PhoP in the limit $t \to \infty$, and $r = 1$ min^{-1} is the exponential decay rate.

The signal intensity for the second input (due to activation of the PmrA/PmrB two-component system) in the models is determined by the rates of phosphorylation and dephosphorylation of PmrA. We distinguish two situations: “strong activation” (higher phosphorylation, lower dephosphorylation rate) and “mild activation” (lower phosphorylation, higher dephosphorylation rate). We assume that both phosphorylation and dephosphorylation are performed by the PmrB protein [23]. At every moment, a fraction $f \in [0,1]$ of all PmrB molecules are in the kinase state, and the fraction $1-f$ are in the phosphatase state (I. Zwir, H. Huang, and E. A. Groisman, *in preparation*). For the FCL, FFL, and the direct regulation circuit, we set $k_p = k_p^{\max} f$, $k_{-p} = k_{-p}^{\max}(1-f)$, where $k_p^{\max}$ and $k_{-p}^{\max}$ are the maximum phosphorylation and dephosphorylation capacities, respectively. For strong activation of the second input, $f = 0.3$; for mild activation, $f = 0.05$.

Parameter sampling procedures for the models

The parameters for the FCL, FFL, and direct regulation circuit were sampled from uniform distributions over the appropriate intervals (Table S2). As described in the main text (Materials and Methods), a pair of parameter sets, one for the FCL and another one for direct regulation circuit, was accepted or rejected depending on whether the model outputs for these models satisfied the filtering criteria. For the FCL and the direct regulation circuit, we considered the case when there is activation of the PhoP input, and calculated the ratio of the output level at the reference time (30 min) and time 0 (activation ratio), both for strong and mild activation of the second input. For the pair of parameter sets to be accepted, all four ratios (2 regulation circuits, 2 states of the second input) were required to exceed the activation threshold (selected to equal 5). The pairs of parameter sets were generated randomly until the number of accepted pairs reached

1000. The pairs of parameter sets for the FFL and the direct regulation circuit were generated in a similar way; however, the filtering criteria were different. It is known that when the second input is inactive, two-component systems connected by a transcriptional cascade cannot be activated [24]. Therefore, we selected only the parameter sets for which, when the second input is under mild activation, at least one of the following conditions holds true for the transcriptional cascade (FFL lacking the direct activation branch): (a) the activation ratio is less than 1.5; (b) the output level at 30 min is over 10 times less than that for strong activation of the second input; (c) output level of the FFL is not greater than 0.005. Other filtering criteria were analogous to those described for the FCL.

In addition to independent parameter generation (Figures 5A–D), we also implemented the "small-noise" strategy of parameter sampling (Figures S4–S6). According to this strategy, after generating a parameter set for the direct regulation circuit, we sampled the FCL parameters from intervals that depended on the values of the generated direct regulation parameters (the sampling distribution was uniform). For example, if the parameter value was $x > 0$, the value for the equivalent FCL parameter was sampled from the interval $[x - \alpha x, x + \alpha x]$, where $\alpha \in [0, 1]$ was the "noise level". If $\alpha = 0$, the small-noise scheme reduces to the no-noise scheme. We considered the cases $\alpha = 0$, $\alpha = 0.3$, and $\alpha = 0.95$. The FCL parameters that do not have equivalents in the direct regulation circuit were sampled randomly (Table S2) and independently. Filtering was performed as above; the case of FFL was treated analogously. The results obtained by small-noise simulations (Figures S4–S6) were qualitatively similar to the ones for independent parameter generation (see main text and Figures 5A–D).

The ODEs for the models with sampled and filtered parameters were solved numerically using MATLAB's ode15s function with relative tolerance 1×10^{-6} and absolute tolerance 1×10^{-9}.

These trajectories were used to calculate the activation and deactivation delays whose distributions are plotted in Figures 5A–D and Figures S4–S6.

Effect of the absence of the direct regulation branch in the FCL and FFL

In the versions of our models with no direct *pbgP* control by PhoP, we set the corresponding affinity (K_2) equal to 0. Such a modification necessarily leads to a decrease in the output levels; we shall prove this statement for the FCL, and the FFL model can be treated in a similar way. Equation 1 with $K_2^{fcl} > 0$ can be written in the form

$$\frac{dX_1}{dt} = F(t) - k_{-pbgP}^{fcl} X_1, \tag{12}$$

where $F(t)$ does not depend on $X_1(t)$. The function $F(t)$ can be found by solving Equations 2–5 independently of Equation 1. If we set $K_2^{fcl} = 0$, we obtain the following equation for X_1:

$$\frac{d\tilde{X}_1}{dt} = \tilde{F}(t) - k_{-pbgP}^{fcl} \tilde{X}_1, \tag{13}$$

where

$$\tilde{F}(t) < F(t) \tag{14}$$

(because $[\text{PhoP-P}] > 0$). The solution to Equation 12 is given by the well-known formula

$$X_1(t) = X_1(0)\exp\left(-k_{-pbgP}^{fcl}\, t\right) + \int_0^t \exp\left(-k_{-pbgP}^{fcl}(t-s)\right) F(s)\,ds\,;$$

similarly,

$$X_1(t) = \tilde{X}_1(0)\exp\left(-k_{-pbgP}^{fcl}\, t\right) + \int_0^t \exp\left(-k_{-pbgP}^{fcl}(t-s)\right) \tilde{F}(s)\,ds\,.$$

We consider the situation when $X_1(0)$ and $\tilde{X}_1(0)$ are the steady-state concentrations corresponding to the level of $[\mathrm{PhoP\text{-}P}]$ at time 0. Setting the derivatives in Equations 12–13 to 0, we obtain that

$$X_1(0) = F(0)/k_{-pbgP}^{fcl}, \qquad \tilde{X}_1(0) = \tilde{F}(0)/k_{-pbgP}^{fcl};$$

therefore,

$$X_1(0) > \tilde{X}_1(0). \tag{15}$$

As a consequence of Equation 14, we also have

$$\int_0^t \exp\left(-k_{-pbgP}^{fcl}(t-s)\right)F(s)ds > \int_0^t \exp\left(-k_{-pbgP}^{fcl}(t-s)\right)\tilde{F}(s)ds \quad \text{for} \quad t>0.$$

This, in combination with Equation 15, proves

$$X_1(t) > \tilde{X}_1(t). \tag{16}$$

It is easy to see that this inequality also holds if $X_1(0) = \tilde{X}_1(0)$ (and $X_1(0)$, $\tilde{X}_1(0)$ are not necessarily steady-state concentrations).

References

1. Janes BK, Bender RA (1998) Alanine catabolism in *Klebsiella aerogenes*: Molecular characterization of the dadAB operon and its regulation by the nitrogen assimilation control protein. J Bacteriol 180: 563-570.

2. Snavely MD, Miller CG, Maguire ME (1991) The Mgtb Mg^{2+} transport locus of *Salmonella typhimurium* encodes a P-type ATPase. J Biol Chem 266: 815-823.

3. Datsenko KA, Wanner BL (2000) One-step inactivation of chromosomal genes in *Escherichia coli* K-12 using PCR products. Proc Natl Acad Sci USA 97: 6640-6645.

4. Bochner BR, Huang HC, Schieven GL, Ames BN (1980) Positive selection for loss of tetracycline resistance. J Bacteriol 143: 926-933.

5. Kato A, Mitrophanov AY, Groisman EA (2007) A connector of two-component regulatory systems promotes signal amplification and persistence of expression. Proc Natl Acad Sci USA 104: 12063-12068.

6. Kox LFF, Wösten MMSM, Groisman EA (2000) A small protein that mediates the activation of a two-component system by another two-component system. EMBO J 19: 1861-1872.

7. Winfield MD, Groisman EA (2004) Phenotypic differences between *Salmonella* and *Escherichia coli* resulting from the disparate regulation of homologous genes. Proc Natl Acad Sci USA 101: 17162-17167.

8. Bell KS, Sebaihia M, Pritchard L, Holden MTG, Hyman LJ, et al. (2004) Genome sequence of the enterobacterial phytopathogen *Erwinia carotovora* subsp *atroseptica* and characterization of virulence factors. Proc Natl Acad Sci USA 101: 11105-11110.

9. Blattner FR, Plunkett G, Bloch CA, Perna NT, Burland V, et al. (1997) The complete genome sequence of *Escherichia coli* K-12. Science 277: 1453-&.

10. Duchaud E, Rusniok C, Frangeul L, Buchrieser C, Givaudan A, et al. (2003) The genome sequence of the entomopathogenic bacterium *Photorhabdus luminescens*. Nature Biotechnol 21: 1307-1313.

11. Wei J, Goldberg MB, Burland V, Venkatesan MM, Deng W, et al. (2003) Complete genome sequence and comparative genomics of *Shigella flexneri* serotype 2a strain 2457T. Infect Immun 71: 2775-2786.

12. Deng W, Burland V, Plunkett G, Boutin A, Mayhew GF, et al. (2002) Genome sequence of *Yersinia pestis* KIM. J Bacteriol 184: 4601-4611.

13. Thompson JD, Gibson TJ, Plewniak F, Jeanmougin F, Higgins DG (1997) The CLUSTAL X windows interface: flexible strategies for multiple sequence alignment aided by quality analysis tools. Nucleic Acids Res 25: 4876-4882.

14. Gunn JS, Lim KB, Krueger J, Kim K, Guo L, et al. (1998) PmrA-PmrB-regulated genes necessary for 4-aminoarabinose lipid A modification and polymyxin resistance. Mol Microbiol 27: 1171-1182.

15. Kato A, Tanabe H, Utsumi R (1999) Molecular characterization of the PhoP-PhoQ two-component system in *Escherichia coli* K-12: Identification of extracellular Mg^{2+}-responsive promoters. J Bacteriol 181: 5516-5520.

16. Lejona S, Aguirre A, Cabeza ML, Vescovi EG, Soncini FC (2003) Molecular characterization of the Mg^{2+}-responsive PhoP-PhoQ regulon in *Salmonella enterica*. J Bacteriol 185: 6287-6294.

17. Yamamoto K, Ogasawara H, Fujita N, Utsumi R, Ishihama A (2002) Novel mode of transcription regulation of divergently overlapping promoters by PhoP, the regulator of two-component system sensing external magnesium availability. Mol Microbiol 45: 423-438.

18. Kato A, Latifi T, Groisman EA (2003) Closing the loop: The PmrA/PmrB two-component system negatively controls expression of its posttranscriptional activator PmrD. Proc Natl Acad Sci USA 100: 4706-4711.

19. Marchal K, De Keersmaecker S, Monsieurs P, van Boxel N, Lemmens K, et al. (2004) *In silico* identification and experimental validation of PmrAB targets in *Salmonella typhimurium* by regulatory motif detection. Genome Biol 5: R9.

20. Wösten MMSM, Groisman EA (1999) Molecular characterization of the PmrA regulon. J Biol Chem 274: 27185-27190.

21. Meir E, Munro EM, Odell GM, Von Dassow G (2002) Ingeneue: A versatile tool for reconstituting genetic networks, with examples from the segment polarity network. J Experim Zool 294: 216-251.

22. Shin D, Lee J, Huang H, Groisman EA (2006) A positive feedback loop promotes transcription surge that jump-starts *Salmonella* virulence circuit. Science 314: 1607-1609.

23. Kato A, Groisman EA (2004) Connecting two-component regulatory systems by a protein that protects a response regulator from dephosphorylation by its cognate sensor. Genes Dev 18: 2302-2313.

24. Bijlsma JJE, Groisman EA (2003) Making informed decisions: regulatory interactions between two-component systems. Trends Microbiol 11: 359-366.
